# Neurological Adverse Effects in Patients of Advanced Colorectal Carcinoma Treated with Different Schedules of FOLFOX

**DOI:** 10.1155/2013/379870

**Published:** 2013-09-29

**Authors:** Nusrat Bano, Rahila Najam, Ahmed Mateen

**Affiliations:** ^1^Department of Pharmacology, University of Karachi, Karachi 75270, Pakistan; ^2^Department of Pharmacy, Jinnah University for Women, Karachi 74600, Pakistan; ^3^Ziauddin College of Pharmacy, Ziauddin University, 4/B, Block 6, Shahrah-e-Ghalib, Clifton, Karachi 75600, Pakistan; ^4^Karachi Institute of Radiotherapy and Nuclear Medicine (KIRAN), Karachi 75330, Pakistan

## Abstract

The study is designed to assess the frequency and severity of few dose limiting neurological adverse effects of four different schedules of FOLFOX. Patients with histologically confirmed advanced colorectal carcinoma (CRC) were included in the study. Toxicity was graded according to CTC v 2.0. The frequency of grade 3 and 4 adverse effects was comparatively assessed in each treatment arm. The difference in the pattern of toxicity between the treatment schedule was evaluated. The most frequent adverse symptom of neurological adverse effect was grade 1 paresthesia in the patients treated with FOLFOX4 schedule. Grade 4 peripheral neuropathy was reported in few patients of FOLFOX7 treatment arm. Frequency and onset of neurological adverse effects like paresthesia, dizziness, and hypoesthesia were significantly different (*P* < 0.05), whereas frequency and onset of peripheral neuropathy were highly significant (*P* < 0.01) in each treatment arm of FOLFOX. Peripheral neuropathy was associated with electrolyte imbalance and diabetes in few patients. Frequency of symptoms, for example, paresthesia, is associated with increased number of recurrent exposure to oxaliplatin (increased number of cycles) even at low doses (85 mg/m^2^), whereas severity of symptoms, for example, peripheral neuropathy, is associated with higher dose (130 mg/m^2^) after few treatment cycles.

## 1. Introduction

Incorporation of Oxaliplatin in 5FU/LV regimen has increased the median overall survival rate and progression free survival in patients of advanced colorectal carcinoma. The most frequent dose limiting toxicity of Oxaliplatin is peripheral neuropathy next to neutropenia. Neurotoxicity of Oxaliplatin is exacerbated as an acute sensory transient response, for example, paresthesia and dysesthesia in hand, feet, and peri oral area [1], which appears during or after exposure to Oxaliplatin. Sensory neurotoxicity with oxaliplatin is progressive, cumulative, and reversible, often manifested as delayed effects (12 to 18 months after discontinuation of the therapy). Peripheral neuropathy is hence regarded as the main “safety concern” for chemotherapy with Oxaliplatin, evident as frequent distal, transient paresthesia within the first few minutes of infusion [2]. The cumulative peripheral sensory neuropathy at the total dose of ≊800 mg/m^2^ requires cessation of therapy [3]. Acute syndrome of neurotoxicity is evident in 1-2% of patients shortly after the infusion, whereas the chronic syndrome is manifested as a dose limiting toxicity in 12–15% patients at the cumulative dose of 780–850 mg/m^2^ [4]. The platinum derivative drugs have molecular affinity for the peripheral nervous system [5, 6], and thus, Oxaliplatin induced peripheral neuropathy is due to the damage imparted to the peripheral sensory neurons [7, 8], leading to the impairment of peripheral neuronal dysfunction [9, 10]. Chronic Oxaliplatin treatment causes a decrease in the conduction velocity in the digital and caudal nerves leading to associated decrease in caudal action potential aptitude [11]. Oxaliplatin causes a “decrease in phosphorylated neurofilaments in DRG neurons with concomitant alterations in sensory axon” that leads to decrease in the diameter of DRG neuronal cell bodies and indicates neuronal atrophy [12]. Certain gene polymorphisms are identified as predisposing factors for peripheral sensory neuropathy [13, 14]. The pathology of peripheral neuropathy is difficult to be defined by nerve conduction studies [15]. Oxaliplatin induces a direct effect on the excitation potential of sensory neurons and muscle cells. Gamelin et al. (2007) reported that the key components of oxalate synthesis pathways are associated with neurotoxicity, and a minor haplotype in AGXT was able to predict acute and chronic toxicity [14]. The sensitive axonal excitability technique shows that neuronal sodium channel dysfunction is associated with the etiology of CINP [10].  Table 1 comprises of reported phase II and III studies, showcasing the outcome of interventions employed to manage oxaliplatin induced neurotoxicity.

## 2. Patients and Methods

The study was designed in the Department of Pharmacology, University of Karachi, and was conducted in a leading cancer hospital in Pakistan, following institutional authorization, on the patients being admitted during 2009–2012, following informed patients consent. Inclusion criteria were maintained on the following grounds.

1. Histologically confirmed advanced colorectal carcinoma.
2. Adequate blood count before therapy.
3. Age 20–80 years.
4. ECOG score of ≤3.
5. No active gastric ulcer and gastrointestinal bleeding (since a year).

Forty-five patients were initially included, and 38 patients were assessable and evaluable by the end of the study. The general patient characteristics are shown in Table 1. The toxicity was graded according to CTC v2.0 on a scale of 1–5 according to the general grade definition of CTC v2.0. The signs and symptoms clearly associated with the disease and the disease progression are not graded during screening of treatment related toxicity. Similarly, treatment delivery system malfunction is not graded during therapy related toxic screening. The defined parameters of neurological toxicities in this study are taste disturbances, headache, paresthesia, dizziness, insomnia, hypoesthesia, and peripheral neuropathy, which were clinically evaluated after each treatment cycle in each treatment arm. The different combination regimens of oxaliplatin with 5FU/LV (FOLFOX), taken as investigational study protocols, for toxicological screening were as follows, where treatment cycles are repeated after two weeks.

*FOLFOX4 Treatment Arm [n* = * 13 (147 Cycles)]*

 
*Oxaliplatin*: 85 mg/m^2^ IV on day 1.
 
*5-Fluorouracil*: 400 mg/m^2^ IV bolus, followed by 600 mg/m^2^ IV continuous infusion for 22 hours on days 1 and 2.
 
*Leucovorin*: 200 mg/m^2^ IV on days 1 and 2 as 2-hour infusion before 5-fluorouracil.

*FOLFOX6 Treatment Arm [n* = * 12 (83 Cycles)]*

 
*Oxaliplatin*: 100 mg/m^2^ IV on day 1.
 
*5-Fluorouracil*: 400 mg/m^2^ IV bolus on day 1, followed by 2400 mg/m^2^ IV continuous infusion for 46 hours.
 
*Leucovorin*: 400 mg/m^2^ IV on day 1 as 2-hour infusion before 5-fluorouracil.

*mFOLFOX6 Treatment Arm [n* = * 5 (60 Cycles)]*

 
*Oxaliplatin*: 100 mg/m^2^ IV 2 hrs infusion on day 1.
 
*5-Fluorouracil*: 2000 mg/m^2^ IV continuous infusion on days 1 and 2 for 46 hours.
 
*Leucovorin*: 100 mg/m^2^ 2 hrs infusion on day 1.

*FOLFOX7 Treatment Arm [n* = * 8 (57 Cycles)]*

 
*Oxaliplatin*: 130 mg/m^2^ IV on day 1.
 
*5-Fluorouracil*: 2400 mg/m^2^ IV continuous infusion on days 1 and 2 for 46 hours.
 
*Leucovorin*: 400 mg/m^2^ IV on day 1 as a 2-hour infusion before 5-fluorouracil.

The frequency of grade 3 and grade 4 adverse effects was comparatively assessed with all toxicity grades by paired samples test. Data was analyzed on SPSS version 19, and comparative assessment was made by one way ANOVA test. *P* value less than 0.05 is considered significant and less than 0.01 is considered highly significant, whereas a value less than 0.001 is considered very highly significant.

## 3. Results

**Figure 1:**
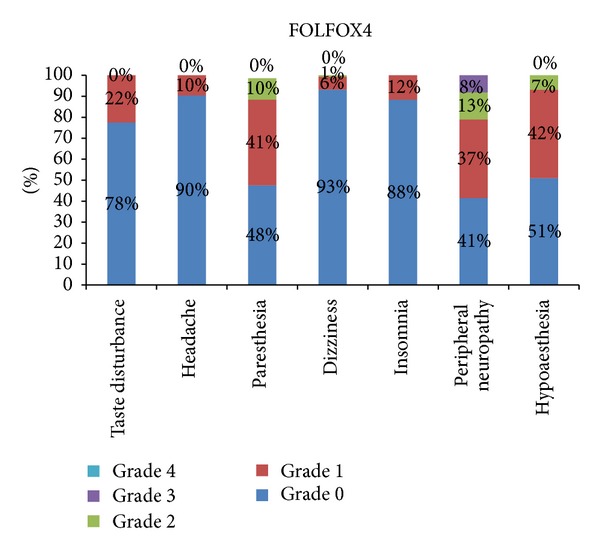
Percentage frequency of neurological adverse effects of all toxicity grades in FOLFOX4 treatment arm.

**Figure 2:**
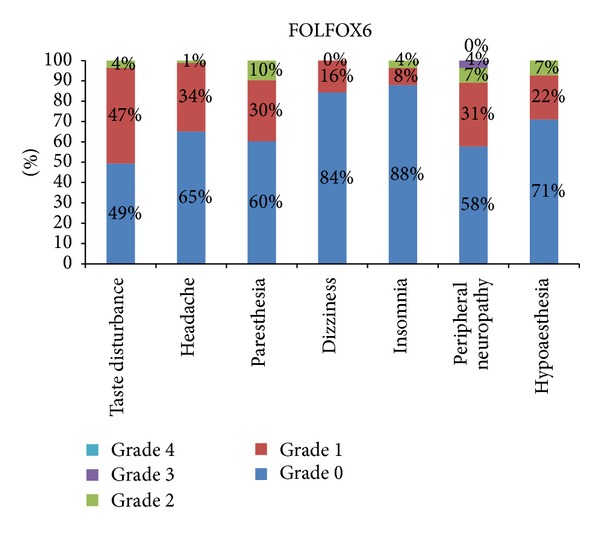
Percentage frequency of neurological adverse effects of all toxicity grades in FOLFOX6 treatment arm.

**Figure 3:**
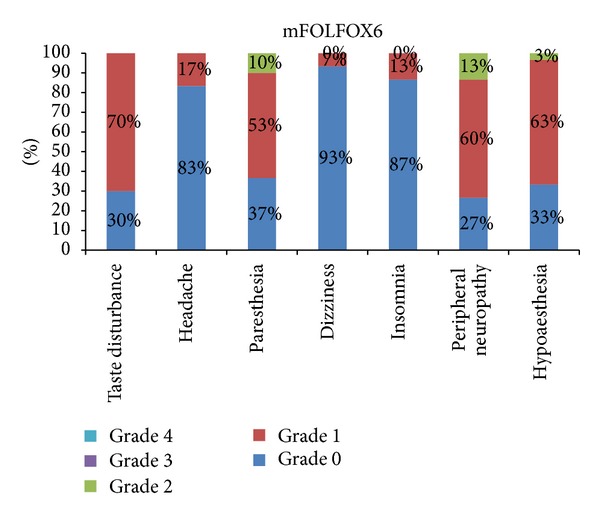
Percentage frequency of neurological adverse effects of all toxicity grades in mFOLFOX6 treatment arm.

**Figure 4:**
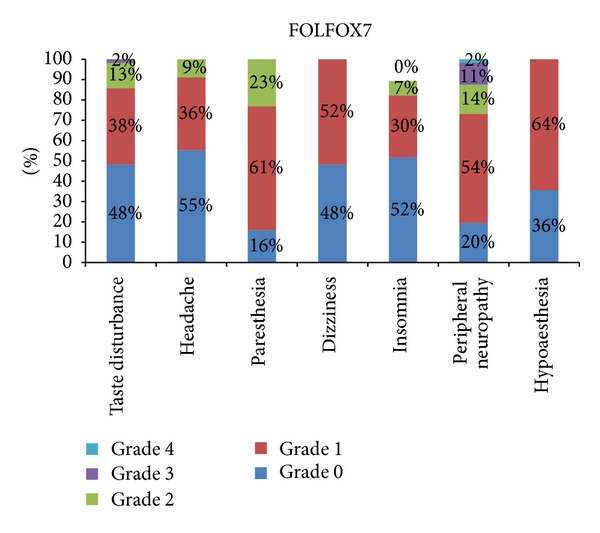
Percentage frequency of neurological adverse effects of all toxicity grades in FOLFOX7 treatment arm.

The most severe symptom reported was peripheral neuropathy 13% grade 2 and 8% grade 3, in patients of FOLFOX4 (Figure 1). The most severe grade of symptoms was grade 3, and the only symptom reported with severity of grade 3 was 4% peripheral neuropathy (Figure 2). The most severe symptom reported in patients of mFOLFOX6 treatment arm is 13% grade 2 peripheral neuropathy. There was no grade 3 or 4 neurological toxicity in patients of mFOLFOX6 arm. The incidence rate of each adverse effect and the severity of the symptoms with related frequency are shown in Figure 3. The most severe symptom reported in patients of FOLFOX7 treatment arm is grade 3 peripheral neuropathy in 11% patients and 2% grade 4 peripheral neuropathy (Figure 4). The difference between the incidence rate of grade 1 and 2 toxicity and grade 3 toxicity of all parameters in neurological toxicity is very highly significant (*P* < 0.001). The difference between grade 3 neurological toxicity with all grades of toxicity is shown in Table 2. The difference between the incidence rate of grade 4 toxicity with all grades of toxicity for each parameters of neurological toxicity is highly significant (*P* < 0.001). The difference between grade 4 neurological toxicity with all grades of toxicity is shown in Table 3. There was no difference in the incidence rate of adverse effects like headache and insomnia between the different schedules of FOLFOX. The frequency and onset of neurological toxic symptoms like paresthesia, dizziness, and hypoesthesia (*P* < 0.05) were significantly different, and peripheral neuropathy (*P* < 0.01) was highly significantly different in each treatment arm of FOLFOX (Table 4).

## 4. Discussion

Peripheral neuropathy is more frequently reported in patients of FOLFOX7 and mFOLFOX6 treatment arms as compared to FOLFOX6 and FOLFOX4 treatment arms. Chemotherapy induced peripheral neuropathy with nociceptive sensory loss during treatment was a very painful condition in some of our patients, although the association between the pain and the loss of sensation is not verified [27, 28]. The most frequent neurological adverse effect reported in the patients of FOLFOX4 is grade 1 paresthesia. Cold sensitive muscle contractions of the jaw and extremities were observed in few patients, giving way to sensory ataxia towards the end of the therapy. Hyponatremia was also assessed in these patients.

The most frequent adverse neurological symptom reported in the patients of FOLFOX6 treatment arm was grade 1 taste disturbance (39 cases) followed by headache (28 cases), whereas the least frequently reported neurological adverse event was insomnia (7 cases). Grade 4 neurological toxicity was not reported in any patient of FOLFOX6 treatment arm. Female patients treated with FOLFOX6 are more prone to risk of peripheral neuropathy and hypoaesthesia. Patients, presented with grade 3 peripheral neuropathy were treated with electrolyte reimbursement who with positive outcome manifesting as reduction in severity of the symptom. The symptoms of CIPN (chemotherapy induced peripheral neuropathy) were also significantly reduced by individualized treatment with calcium/magnesium (Ca/Mg) infusion and vitamin E.

Most frequent adverse symptom of neurological toxicity reported in the patients treated with mFOLFOX was mild taste disturbances (42 cases) at different stages during the course of treatment. Grade 2 hypoesthesia was reported in 7% patients of FOLFOX6 treatment arm. Headache was a mild and less frequent symptom (17%) in mFOLFOX6 patients, whereas peripheral neuropathy, hypoesthesia, and paresthesia were the most frequently reported neurological toxicity in the patients. 

It is important to assess the neurological toxicities in these patients as a delayed toxic effect during followup since the platinum compounds are unique in the sense that they produce ganglionopathy, and the progression of sensory loss may progress even after the cessation of therapy over months referred to as “coating” [28]. The patients experiencing CIPN have no signs of axonal degeneration shown by nerve biopsy study or neurophysiological exams [29, 30]. Glutathione is also shown to be effective in reducing the symptoms of CIPN, whereas agents like topical pain relievers (baclofen/amitriptyline/ketamine gel) and serotonin and norepinephrine reuptake inhibitors (venlafaxine and duloxetine) also have proven efficacy against CIPN [31]. 

The most frequent symptom of neurotoxicity reported in the patients included in FOLFOX7 treatment arm was grade 1 hypoaesthesia (36 cases). Grade 4 peripheral neuropathy was reported only in FOLFOX7 treatment arm, and although the incidence rate of grade 4 peripheral neuropathy was low, but the treatment was delayed and doses of Oxaliplatin reduced to one and then two levels in the patient. Persistence of severity of the symptom required discontinuation of treatment. One of these patients was treated for tuberculosis in the past and was since suffering from previous treatment induced neurological symptoms. There were only few cases of grade 3 peripheral neuropathy and taste disturbances; however, the overall difference between the incidence rate of grade 3 toxicity and grade 1 and 2 toxicity was very highly significant. Grade 4 peripheral neuropathy was reported in FOLFOX7 only. Toxic neuropathy can occur in patients who have preexisting neuropathological disorder such as underlying inherited or inflammatory neuropathies. The selection of a specific schedule of FOLFOX to minimize the risk of symptoms like paresthesia, hypoesthesia, and dizziness is important as the patterns of these toxicities are variable in different schedules of treatment. Severity and frequency of peripheral neuropathy can be carefully avoided by selecting regimens with less toxic neurological manifestations using mFOLFOX6. Our observations during this study support that pretreatment of hypomagnesaemia and anemia conversely associated with age can be identified as predictors of neurotoxicity in oxaliplatin based treatment [32]. Although the use of nutraceuticals, that is, vitamin E, Vitamin B6 and calcium as prophylactic or pretreatment agents for the management of oxaliplatin induced peripheral neuropathy is not well established [33]; few of our patients responded positively to them. A detailed and comprehensive study is further required to confirm the effective and undisputed protocol for the management of oxaliplatin induced neurotoxicity.

## 5. Conclusion

Grade 4 peripheral neuropathy with nociceptive sensory loss was a painful complication reported in a patient in FOLFOX7. Grade 3 peripheral neuropathy was reported in all other schedules of FOLFOX except in the patients treated with modified schedule of FOLFOX6. The incidence rate of paresthesia was higher in schedules with increased number of treatment cycles despite lower dose of Oxaliplatin.

## Figures and Tables

**Table 1:** Management of oxaliplatin induced neurotoxicity comprising of phase II and phase III studies.

Study	Year	Patient type	Treatment type	Intervention	Outcome
Wen et al. [16]	2013	Colorectal cancer (*N* = 1170)	Oxaliplatin based chemotherapy	Ca/Mg infusion	Reduction in grade 3 acute neurotoxicity

Xu et al. [17]	2013	Gastrointestinal cancer (*N* = 1765)	Oxaliplatin based chemotherapy	Ca/Mg infusion	Reduction in grade 1 and 2 and no effect on grade 3 neurotoxicity

Grothey et al. [18]	2013	Colon cancer (*N* = 353)	FOLFOX	Ca/Mg infusion	No reduction in cumulative sensory neurotoxicity

Gobran and Nagy [19]	2013	Colorectal cancer (*N* = 60)	Oxaliplatin based chemotherapy	Ca/Mg infusion	Significant reduction in chemotherapy induced neuropathy

de Afonseca et al. [20]	2013	Colorectal and gastric cancer (*N* = 34)	Oxaliplatin based chemotherapy	Vitamin E	No reduction in chemotherapy induced neuropathy

Grothey et al. [21]	2011	Colon cancer (*N* = 102)	Oxaliplatin, 5 FU, and leucovorin	Ca/Mg infusion	No effect in cold induced sensory neuropathy Effective neuroprotective effect of Ca/Mg therapy in oxaliplatin induced peripheral neuropathy

Knijn et al. [22]	2011	Advanced colon cancer (*N* = 732)	Capecitabine, oxaliplatin, and bevacizumab with and without cetuximab	Ca/Mg infusion	Significant reduction in chemotherapy induced neuropathy

Kottschade et al. [23]	2011	Not specified (*N* = 207)	Taxanes, cisplatin, oxaliplatin, and carboplatin based chemotherapy	Vitamin E	No significant reduction in chemotherapy induced neuropathy

Ishibashi et al. [24]	2010	Metastatic colorectal cancer (*N* = 33)	FOLFOX6	Ca/Mg infusion	No significant reduction in chemotherapy induced neuropathy

Chay et al. [25]	2010	Colorectal cancer (*N* = 27)	FOLFOX4/ Capecitabine + oxaliplatin	Ca/Mg infusion	No significant reduction in chemotherapy induced neuropathy

Gamelin et al. [26]	2004	Colorectal cancer (*N* = 161)	Oxaliplatin based chemotherapy	Ca/Mg infusion	Low frequency of grade 3 distal paresthesia in Ca/Mg group No case of pseudolaryngospasm in Ca/Mg group

**Table 2:** Patient characteristics.

Parameters	FOLFOX4		FOLFOX6		mFOLFOX6		FOLFOX7	
	No. of patients	%	No. of patients	%	No. of patients	%	No. of patients	%
Gender								
Male	10	76.92	9	75	4	80	6	75
Female	3	23.08	3	25	1	20	2	25
Age: year								
Median	63		60		51		67	
Range	58–64		52–68		47–53		49–72	
ECOG performance status								
0	1	7.69	1	8.33	0	0	0	0
1	4	30.77	1	8.33	3	60	2	25
2	7	53.85	10	83.33	2	40	5	62.5
3	1	7.69	0	0	0	0	1	12.5
Primary site								
Colon	10	76.92	7	58.33	2	40	3	37.5
Rectum	3	23.08	5	41.67	1	20	2	25
Multiple	0	0	0	0	2	40	3	37.5
No. of sites								
1	7	53.85	4	33.33	3	60	6	75
≥2	6	46.15	8	66.67	2	40	2	25
AlkPO4								
Normal	3	23.08	6	50	3	60	5	62.5
Increased	7	53.85	3	25	2	40	3	37.5
Unknown	3	23.08	3	25	0	0	0	0

**Table 3:** Comparative differences in frequency of grade 3 neurological adverse effects with grade 1 and grade 2 adverse effects.

Paired samples test						
Toxicity	Mean	Std. deviation	Mean difference	*t *	df	*P *value
Taste disturbance grade 1, 2	3.816	3.525	3.789	6.608	37.000	0.000
Taste disturbance grade 3	0.026	0.162				
Headache grade 1, 2	2.053	2.588	2.053	4.888	37.000	0.000
Headache grade 3	0.000	0.000				
Paresthesia grade 1, 2	5.079	3.529	5.079	8.872	37.000	0.000
Paresthesia grade 3	0.000	0.000				
Dizziness grade 1, 2	1.474	2.345	1.474	3.874	37.000	0.000
Dizziness grade 3	0.000	0.000				
Insomnia grade 1, 2	1.474	2.357	1.474	3.855	37.000	0.000
Insomnia grade 3	0.000	0.000				
Peripheral neuropathy grade 1, 2	4.947	3.385	4.395	7.045	37.000	0.000
Peripheral neuropathy grade 3	0.553	1.589				
Hypoaesthesia grade 1, 2	4.526	4.105	4.526	6.797	37.000	0.000
Hypoaesthesia grade 3	0.000	0.000				

*P* value < 0.05 (significant), *P* value < 0.01 (highly significant), and *P* value < 0.001 (very highly significant).

**Table 4:** Comparative differences in frequency of grade 4 neurological adverse effects with all grades of toxicity.

Paired samples test						
Toxicity	Mean	Std. deviation	Mean difference	*t *	df	*P* value
Taste disturbance grade 1, 2, and 3	3.842	3.522	3.842	6.724	37.000	0.000
Taste disturbance grade 4	0.000	0.000				
Headache grade 1, 2, and 3	2.053	2.588	2.053	4.888	37.000	0.000
Headache grade 4	0.000	0.000				
Paresthesia grade 1, 2, and 3	5.079	3.529	5.079	8.872	37.000	0.000
Paresthesia grade 4	0.000	0.000				
Dizziness grade 1, 2, and 3	1.474	2.345	1.474	3.874	37.000	0.000
Dizziness grade 4	0.000	0.000				
Insomnia grade 1, 2, and 3	1.474	2.357	1.474	3.855	37.000	0.000
Insomnia grade 4	0.000	0.000				
Peripheral neuropathy grade 1, 2, and 3	5.500	3.630	5.474	9.277	37.000	0.000
Peripheral neuropathy grade 4	0.026	0.162				
Hypoaesthesia grade 1, 2, and 3	4.526	4.105	4.526	6.797	37.000	0.000
Hypoaesthesia Grade 4	0.000	0.000				

*P* value < 0.05 (significant), *P *value < 0.01 (highly significant), and *P* value < 0.001 (very highly significant).

**Table 5:** Comparative differences in incidence rate of neurological adverse effects between each treatment arm of FOLFOX.

ANOVA		
Toxicity	*F *	*P* value
Neurological		
Taste disturbance	4.370	0.010
Headache	1.169	0.336
Paresthesia	3.475	0.026
Dizziness	3.458	0.027
Insomnia	0.955	0.425
Peripheral neuropathy	5.077	0.005
Hypoaesthesia	3.598	0.023

*P* value < 0.05 (significant), *P* value < 0.01 (highly significant), and *P* value < 0.001 (very highly significant).
